# All-Inside Anterior Cruciate Ligament Reconstruction Technique: Tips and Tricks

**DOI:** 10.3390/jcm12185793

**Published:** 2023-09-06

**Authors:** Francesco Bosco, Fortunato Giustra, Alessandro Ghirri, Giorgio Cacciola, Alessandro Massè, Marcello Capella

**Affiliations:** 1 Department of Orthopaedics and Traumatology, University of Turin, Centro Traumatologico Ortopedico (CTO), 10124 Turin, Italy; fortunato.giustra@gmail.com (F.G.); dr.cacciola@gmail.com (G.C.); alessandro.masse@unito.it (A.M.); marcello.capella@alice.it (M.C.); 2 Department of Orthopaedics and Traumatology, Ospedale San Giovanni Bosco di Torino—ASL Città di Torino, 10154 Turin, Italy; alessandro.ghirri@gmail.com

**Keywords:** anterior cruciate ligament, ACL, anterior cruciate ligament reconstruction, ACLR, all-inside, knee, arthroscopy, surgical technique, technical note

## Abstract

The all-inside anterior cruciate ligament reconstruction (ACLR) technique was developed to improve patient outcomes by reducing the procedure’s invasiveness, minimizing complications and pain, and enabling faster postoperative recovery. This study presents a detailed description of the all-inside ACLR technique, which involves the use of quadrupled semitendinosus (ST) graft and suspension devices at both tibial and femoral sites, as well as valuable tips for avoiding complications that may arise during the procedure. The surgical procedure employs retrograde drills to create bony sockets for graft passage, which are then fixed with suspension devices at both the tibial and femoral sites. This technique has no specific restrictions and may be applied to all patients with anterior cruciate ligament (ACL) injuries. The literature reports the advantages, good clinical outcomes, and medium- to long-term graft survival achieved with the all-inside ACLR technique. However, the complications and disadvantages associated with the technique must be considered.

## 1. Introduction

Anterior cruciate ligament reconstruction (ACLR) is one of the most common procedures performed in orthopedic surgery; it is estimated that in the United States alone, 400,000 ACLRs are performed annually [1]. Over the years, several innovations in surgical technique and hardware were developed to combine anatomical ACLR with minimally invasive approaches to reduce bone loss, bleeding, and pain and facilitate a faster rehabilitation program [1,2,3,4,5]. Among the different ACLR procedures, the all-inside technique described by Morgan et al. in 1985 [6] and then improved by Lubowitz et al. in 2011 [7] is gaining popularity as it reflects the criteria of anatomical socket positioning, the reduction of postoperative pain and edema, less invasive hardware, better graft tensioning control, and cortical bone preservation [4,5]. This procedure is characterized by bone half-tunnel creation with retrograde drills and graft fixation with suspension devices at the tibial and femoral sites [5,7]. The graft length required is shorter than other ACLR techniques; the most widely used grafts are the quadrupled ipsilateral semitendinosus (ST) autograft or quadriceps tendon [4,5]. Moreover, the all-inside ACLR technique preserves the “bone stock” by creating half-tunnels, using tendons without bony attachments and bone scaffolds [8,9].

The all-inside ACLR technique, unlike other ACLR procedures, has no significant restriction and may be applied to all patients with an anterior cruciate ligament (ACL) rupture, including adolescents, due to growth cartilage preservation performing sockets entirely within the epiphysis; this approach reduces bone growth damage risk and postoperative pain [8,9,10]. The all-inside ACLR procedure has proven to be safe and successful, as was demonstrated in several mid- to long-term studies that evaluated both clinical outcomes and neo-ACL rupture rates compared with other ACLR procedures [9,10,11,12].

Therefore, several previous studies have comprehensively evaluated the all-inside ACLR technique, analyzing the clinical outcomes, graft rupture rates, and rehabilitation protocols [8,9,10,11,12]. While these studies have made significant contributions, there is ongoing potential for advancing new instrumentation and refined techniques to optimize this surgical approach and provide additional clinical advantages. The aim of our study is to provide new insights to improve outcomes further and reduce the complications associated with this surgical technique by giving some helpful “tips and tricks” based on our clinical experience.

The study aims to describe our all-inside ACLR technique, which uses a quadrupled ST graft fixed with both tibial and femoral suspension devices, to provide procedure standardization and share our tips and tricks to avoid the complications that may occur with this ACLR technique.

## 2. Surgical Technique

### 2.1. Patient Positioning and Preoperative Setting

**Figure 1:**
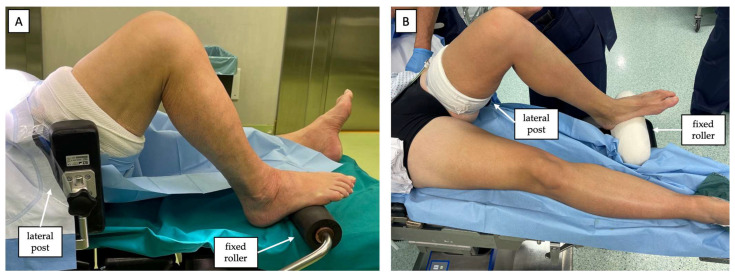
The knee is positioned at 90°, with a lateral post just proximal to the thigh and a fixed roller under the foot. (**A**) Right knee from a lateral point of view. (**B**) Left knee from a medial point of view.

A thorough clinical examination under anesthesia is performed before positioning the patient on the operating table to confirm anteroposterior (AP) knee instability using the Lachman and Anterior Drawer test. Rotational stability is also assessed by performing the pivot shift test and varus/valgus stress to rule out other ligamentous injuries [13]. The patient is then placed in supine decubitus with a tourniquet proximal to the thigh of the operated limb. A lateral post just proximal to the thigh and a fixed roller under the foot are placed to keep the knee at 90°. An additional retaining pad is placed at the contralateral hip to lock the tilting movements of the pelvis during the stress maneuver required to evaluate the medial knee compartment. Finally, the contralateral limb is left free distally (Figure 1).

### 2.2. Graft Harvesting

The surgical procedure begins with graft harvesting and preparation on a dedicated workstation by an assistant. Several tendons can be harvested as grafts, including the ST, gracilis, quadriceps, peroneus longus, and allografts [14,15,16]. Our technique reports the harvesting and preparation of the quadrupled ST graft.

The surgical procedure begins with an incision positioned 2 cm medial to the anterior tibial tuberosity and 4 cm distal to the joint line. A planar dissection is performed and arrives at the sartorius fascia, which is incised to identify the hamstrings. With the aid of an open tendon stripper, only the ST is harvested. The graft is cleaned and bathed with saline solution supplemented with 1 g vancomycin powder and is then placed on a workstation for subsequent graft preparation. The gracilis is usually retained unless the size of the ST is inadequate or has been prematurely amputated inadvertently.

### 2.3. Graft Preparation

**Figure 2:**
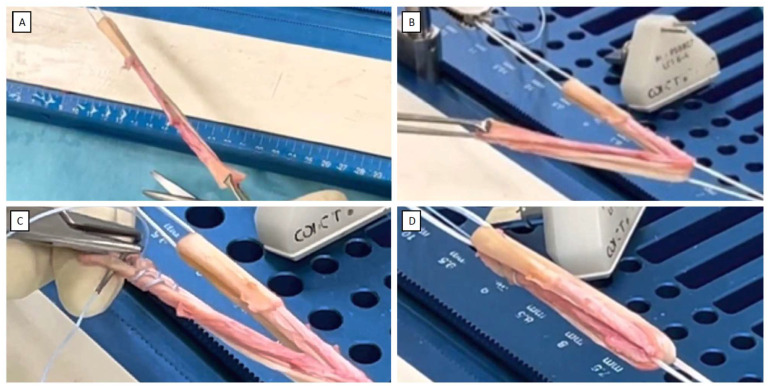
Graft preparation on a workstation. (**A**) The ST graft is duplicated by passing through the loop of a suspension device. (**B**) The ST graft is then quadrupled, using the loop of a second suspension device. (**C**) The free ends of the graft are sutured together with a high-strength multi-strand suture. (**D**) The graft is attached to the workstation clamps under tension.

Graft preparation is performed on a dedicated workstation. The length of the harvested ST should be about 26–28 cm, while the length of the quadrupled ST graft should be about 60–70 mm, and the thickness should be 8–10 mm. The ST graft is first duplicated by passing it across an adjustable-loop suspension device (UltraButton™, Smith&Nephew, Singapore) (Figure 2A). Then, the ST graft is quadrupled by passing it through a second adjustable-loop suspension device (UltraButton™, Smith&Nephew) (Figure 2B), and the free ends of the graft are sutured together with a high-strength multi-strand suture (FiberWire^®^ No. 2; Arthrex, Naples, FL, USA) while keeping the two suture tails free (Figure 2C). FiberWire^®^ is characterized by its high strength, durability, low profile, and ease of handling, making it a preferred choice for ACLR. However, other high-strength multi-strand sutures may also perform effectively in the described procedures. The graft is then attached to the workstation clamps in a tensioned state (Figure 2D).

**Figure 3:**
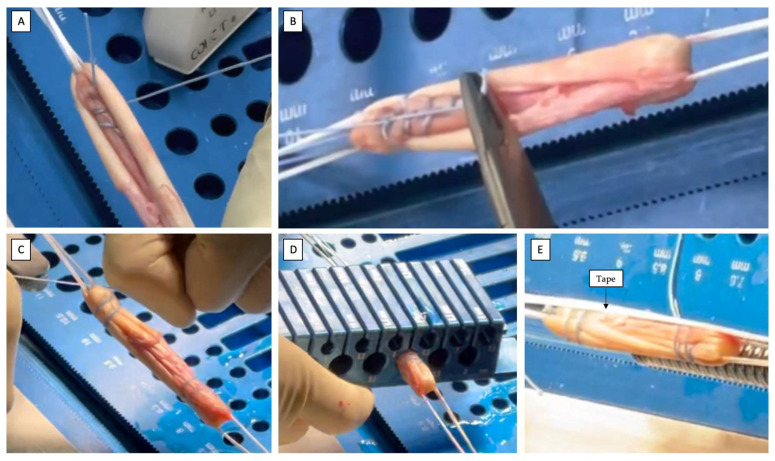
Graft preparation on a workstation. (**A**,**B**) Graft tubularization is performed using high-strength multi-strand sutures, which annularly secure the graft with multiple loops and create an annular tag at a predefined distance. (**C**) Knot the two ends of the sutures used to secure the free ends of the duplicate ST. (**D**) Measure the length and diameter of the quadrupled ST graft using a sizing block. (**E**) Graft prepared, tensioned, and augmented with a suture tape.

Subsequent graft tubularization is performed using a high-strength multi-strand suture (FiberWire^®^ No. 0; Arthrex), which annularly fixes the graft using multiple loops and creates an annular tag at a predefined distance of 15 mm (if the graft has a total length of 60 mm) or 20 mm (if the graft has a total length of 65 mm) from both ends of the graft (Figure 3A,B). Then, a clinician secures the graft by knotting the two ends of the high-strength multi-strand sutures FiberWire^®^ No. 2 that was previously used to stitch the free ends of the duplicated ST (Figure 3C). The length and diameter of the quadrupled ST graft are measured by passing both the femoral and tibial graft extremities into the sizing block (Figure 3D).

**Tips.**  *Annular graft fixation with high-strength multi-strand sutures at defined distances allows accurate assessment of the amount of graft needed for engaging the femoral and tibial socket inside the joint. Assuming a maximum intra-articular length of 25 mm, a typical amount of graft in the femoral and tibial socket is 20 mm for both sides.*

**Tips.**  *Mark the distance of the femoral tunnel at the level of the femoral adjustable-loop suspension device and tag the middle of the graft with an annular mark. This will allow these landmarks to be used to evaluate proper femoral button flipping and intra-articular graft position.*

### 2.4. Internal Brace

If an internal brace is performed, a suture tape (FiberTape^®^, Arthrex) is passed through the femoral buttonholes of the adjustable-loop suspension device (UltraButton™, Smith&Nephew), leaving the tails free at the level of the tibial fixation device (Figure 3E).

### 2.5. Starting Arthroscopic Setting

During graft preparation, the leading surgeon proceeds with the arthroscopic set-up. A diagnostic knee arthroscopy is performed, any accessory tissue lesions found are treated, and the femoral and tibial footprints are identified. Standard anterolateral (AL) and anteromedial (AM) arthroscopic portals are used [17].

**Tips.**  *Perform careful Hoffa’s fat pad debridement for adequate articular visualization.*

### 2.6. Femoral Socket

**Figure 4:**
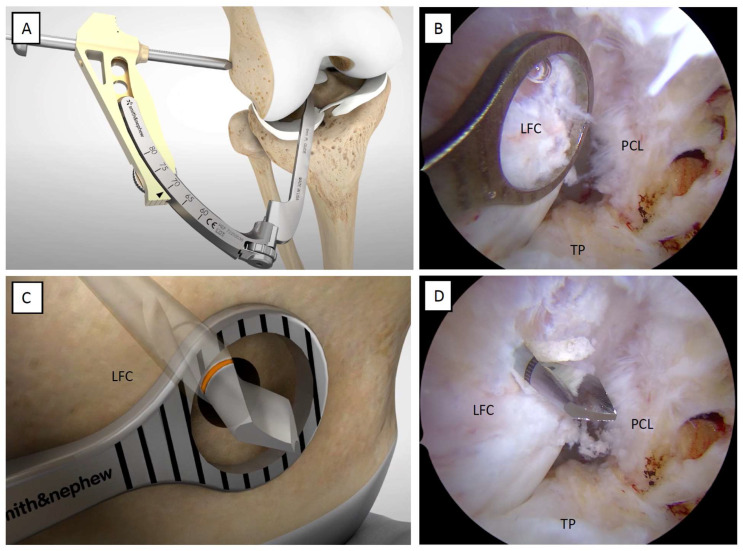
Determining the appropriate ACL femoral footprint. (**A**,**B**) Place the guide of the outside-in aimer arm in the intra-articular site at the center of the native ACL femoral footprint. (**C**) Drill the outer lateral femoral cortical with the guide wire until it exits the center of the intra-articular guide. (**D**) A black mark on the guide wire tip identifies the correct wire penetration. LFC: lateral femoral condyle; PCL: posterior cruciate ligament; TP: tibial plateau.

The AM portals should be used as viewing portals to visualize the ACL femoral footprint better. With the knee positioned in 90° of flexion and the scope in the AM portal, the clinician inserts the outside-in aimer arm through the AL portal and places the head of the guide over the center of the native ACL femoral footprint, which is identified by taking the lateral intercondylar ridge and the lateral bifurcate ridge as a reference point [4,8]. The clinician ensures that the correct outside-in aimer arm has been selected, with the left aimer arm for the left knee and the right aimer arm for the right knee. Once the guide of the outside-in aimer arm is placed at the intra-articular position, the bullet is inserted and secured on the distal lateral femur above the lateral epicondyle. A femoral skin incision is made, ensuring proper bullet seating on the femoral bone (Figure 4A).

**Tips.**  *In case the footprint is not optimally visualized, the presence of a ligamentous remnant or narrow intercondylar gap is strongly suggested to establish a third portal, the accessory anteromedial portal (AAM), which provides good arthroscopic visualization of the femoral footprint, to make an accurate femoral tunnel.*

**Tips.**  *The aiming arm on the femoral side should be set at about 80 degrees, with a variability of about 5 to 10 degrees, depending on the lateral femoral condyle’s anatomical conformation, the soft tissues’ thickness, and the knee’s intercondylar notch depth. This aimer arm positioning allows easy visualization of the correct button flipping, checked by direct arthroscopic viewing using the AL portal as the entry point, and positioning of the scope at the lateral subcutaneous layer. Attempt to respect the femoral entry point of the guidewire, located approximately 2.5 cm proximal to the lateral femoral epicondyle, being careful not to stand too far anterior so as not to compromise the trochlear cartilage.*

**Figure 5:**
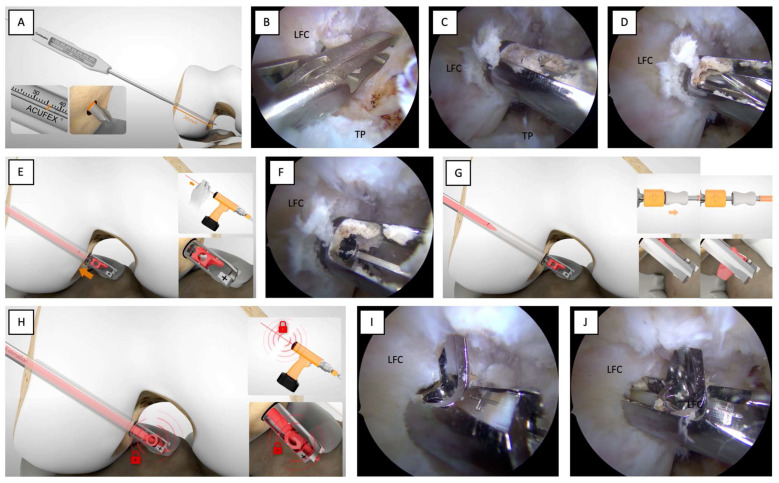
Femoral socket setup using a guide wire and forward movement of the retrograde drill. (**A**) Use a cannulated measuring device to estimate the length of the femoral tunnel. (**B**) During the anterograde drill advancement, use a protective instrument such as a suture retriever/tissue grasper. (**C**,**D**) Perform forward and backward guide-wire movement to remove bone debris embedded in the reamer tip. (**E**,**F**) Retract the guide wire so it is no longer inside the reamer tip. (**G**) Press the handle switch to flip the reamer tip to the retrograde drilling position. (**H**,**I**) The guide wire is again advanced inside the reamer tip to lock the drill into the retrograde drilling configuration. (**J**) Activate the drill with the reamer tip slightly detached from the ACL femoral footprint. LFC: lateral femoral condyle; TP: tibial plateau.

The surgeon drills the outer lateral femoral cortical with the guide wire through the bullet until it exits the center of the intra-articular guide (Figure 4B,C). Direct arthroscopic visualization of the guide ensures that the guide wire is not drilled too far into the joint. Once drilled with the guide wire, the bullet and aimer arm are removed from the joint, leaving only the guide wire in situ. A black mark on the tip of the guide wire identifies the correct wire penetration (Figure 4D). The assistance of a dedicated cannulated measuring device allows the length of the femoral tunnel to be assessed (Figure 5A). Then, the surgeon proceeds with an anterograde progression of the retrograde drill (Trunav™, Smith&Nephew) to its intra-articular ingress. The correct reamer tip size of the retrograde reamer should match the measured graft thickness. Progressive sizes of 0.5 mm may be used.

**Tips.**  *To avoid damaging other intra-articular structures, a protective instrument such as a suture retriever/tissue grasper (KingFisher^®^, Arthrex) should be used during the anterograde drill advancement (Figure 5B).*

Forward and backward guide-wire movement removes the bony debris embedded in the reamer tip (Figure 5C,D). It proceeds by slightly retracting the guide wire so that it is no longer within the reamer tip (Figure 5E,F). A handle switch flips the reamer tip into the retrograde drilling position (Figure 5G). The guide wire is again advanced inside the reamer tip to lock the cutter into the retrograde drilling position configuration (Figure 5H,I).

**Tips.**  *Do not start by activating the drill with the reamer tip when in direct contact with the bone because this may lead to breakage of the reamer tip; therefore, start the drill with the reamer tip slightly detached from the ACL femoral footprint (Figure 5J).*

**Figure 6:**
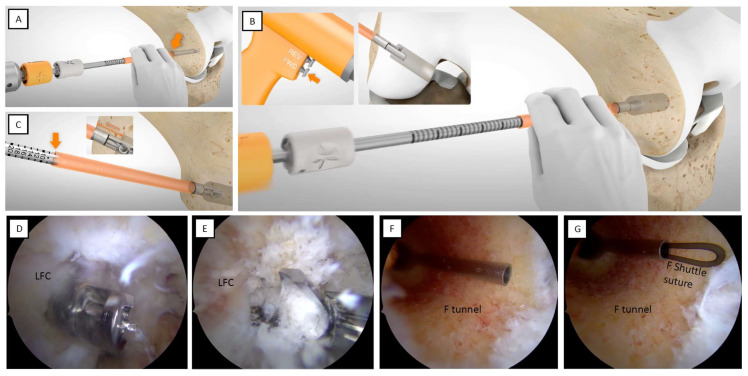
The femoral socket is completed using the retrograde drill. (**A**) Advance the drilling sleeve to the outer lateral femoral cortical. (**B**–**D**) The femoral socket is completed by powering the retrograde reamer by forward drilling with traction to the previously defined measurement. (**E**) Press the handle switch to flip the reamer tip from the retrograde drilling position to the linear drilling position. Remove the guide wire and advance a shuttle suture inside the cannulated retrograde drill. (**F**,**G**) As soon as the shuttle suture is passed through the femoral socket, remove the retrograde drill, leaving only the shuttle suture in place, and retrieve it from the AM portal using a suture retriever/tissue grasper. LFC: lateral femoral condyle; F: femoral.

The drill sleeve is advanced until it encounters the outer lateral femoral cortical to confirm the femoral tunnel measurement that was previously assessed with the dedicated cannulated measuring device (Figure 6A). A femoral socket of the appropriate size is created by powering the retrograde reamer via forward drilling with traction to the previously defined measurement (Figure 6B–D). Then, the retrograde drill is manually advanced into the joint (Figure 6E). The handle switch is pressed to flip the reamer tip from retrograde to linear drilling. The guide wire is removed at this point, and a shuttle suture is advanced inside the cannulated retrograde drill. As soon as the shuttle suture is passed through the femoral socket, the retrograde drill is removed, leaving only the shuttle suture in place, which is retrieved from the AM portal using a suture retriever/tissue grasper (KingFisher^®^, Arthrex) (Figure 6F,G).

**Tips.**  *Always look at the femoral socket to check that it has been correctly created circumferentially and that there are no soft tissues/bone remnants/reamer debris locked inside that could interfere with the graft’s passage. During the drilling phase, it is recommended to position the suction shaver from the AM portal to aspirate the debris from drilling.*

### 2.7. Tibial Socket

For a comprehensive view and to achieve a proper tibial socket, it is recommended that the AL and AAM portals be used for arthroscopic viewing, using the AM portal as the working portal.

**Figure 7:**
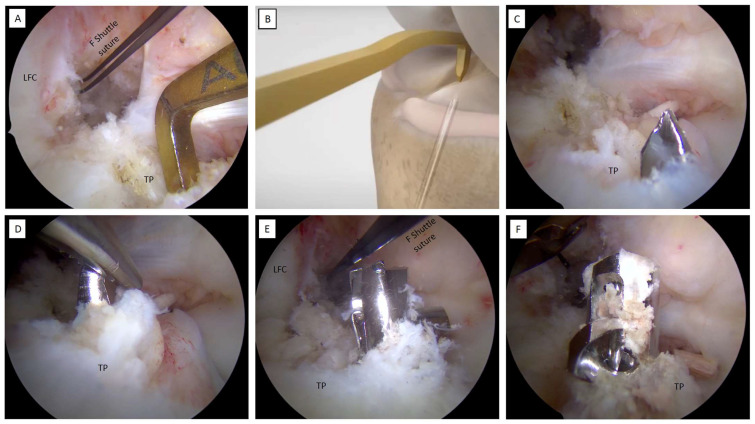
Determining the ACL tibial footprint and drilling the appropriate tibial socket using the retrograde reamer. (**A**,**B**) Place the tip of the ACL Tip Aimer ACUFEX™ Director at the ACL tibial footprint level. (**C**) Advance the guide wire inside the joint. (**D**) Use a suture retriever/tissue grasper during drilling. (**E**,**F**) Perform the same retrograde drilling procedure as is performed at the femoral level. LFC: lateral femoral condyle; F: femoral; TP: tibial plateau.

Through the AM portal, the tip of the ACUFEX™ Director ACL Tip Aimer (Smith&Nephew) is placed at an angle of 55–60° at the level of the ACL tibial footprint, anterior to the posterior border of the anterior horn of the lateral meniscus (Figure 7A,B). The guide position and angle are optimized to maximize the tibial tunnel length, which may be read before drilling by advancing the drill sleeve in contact with the tibial cortical in the same manner as when measuring at the femoral level.

The guide wire is advanced to the inside of the joint (Figure 7C). At this point, a suture retriever/tissue grasper (KingFisher^®^, Arthrex) is placed as protection, and the same retrograde drilling (Trunav™, Smith&Nephew) procedure is performed at the femoral level (Figure 7D–F).

**Tips.**  *It is crucial to have at least 36 mm of tibial tunnel length to create a 30 mm tibial socket while leaving at least 7 mm of cortical bone. This assumes a maximum intra-articular length of 25 mm and approximately 20 mm of graft in the femoral and tibial sockets. Therefore, it is recommended that the femur be drilled to a depth of 20 mm and the tibia drilled to a depth of about 30 mm so that there is an extra 10 mm for subsequent tensioning.*

**Figure 8:**
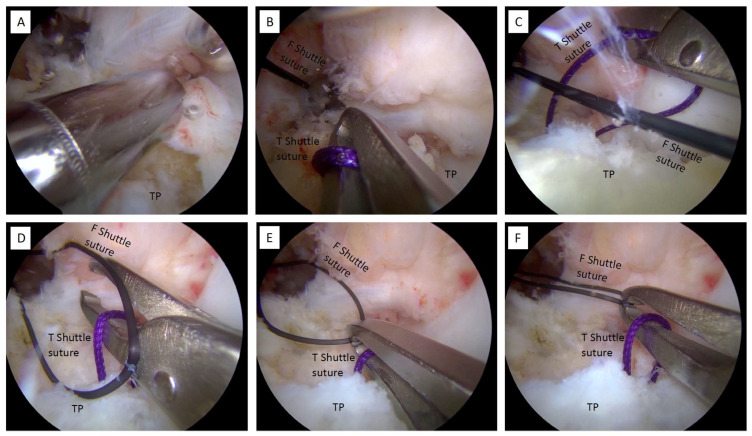
Shuttle sutures. (**A**) Use a suction shaver to aspirate the drilling debris during the drilling phase. (**B**) Use a suture retriever/tissue grasper from the AM portal to catch the shuttle sutures. (**C**,**D**) The proximal section of the jaws slides through the tibial shuttle suture. (**E**,**F**) The distal serrated tip of the jaws is used as a grasper for the femoral shuttle suture. F: femoral; T: tibial; TP: tibial plateau.

**Tips.**  *Always look at the tibial socket to check that it has been created correctly circumferentially and that there are no soft tissues/bone remnants/drill fragments stuck inside that could interfere with the graft passage. During the drilling phase, it is recommended to position the suction shaver from the AL portal to aspirate the debris from drilling (Figure 8A). Use a passing pin and a drill tip of 2.4 mm (Smith&Nephew) with a shuttle suture to pull through the tibial tunnel and retrieve the shuttle suture from the joint using a suture retriever/tissue grasper (KingFisher^®^, Arthrex) (Figure 8B,C).*

### 2.8. Shuttle Sutures

Retract the femoral and tibial shuttle sutures into the joint (Figure 8D). Through the AM portal, retrieve both the shuttle sutures with a suture retriever/tissue grasper (KingFisher^®^, Arthrex) and avoid soft tissue interposition. Specifically, the proximal section of the jaws is used to slide through the tibial shuttle suture, and the distal serrated tip of the jaws is used as a femoral shuttle suture grasper (Figure 8E,F).

**Tips.**  *While retracting the clamp outward from the joint, shuttle sutures must be maintained under tension to keep them apart from each other. This will avoid suture tangle among the femoral and suture shuttles.*

**Tips.**  *Soft silicone cannulas, such as the PassPort Button™ Cannula (Arthrex), are suggested to avoid contact between the graft and the skin.*

**Tips.**  *The AM portal must be dilated to permit graft introduction.*

### 2.9. Femoral Socket Graft Passage

**Figure 9:**
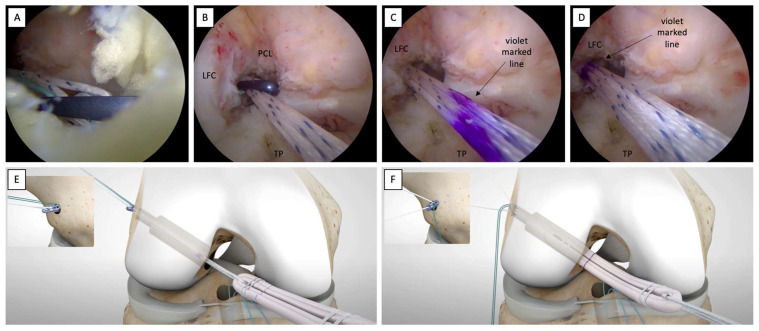
Passage of the femoral button into the femoral socket and fixation. (**A**) Femoral button at the intra-articular level. (**B**) Femoral button entering the femoral socket. (**C**) Violet markings indicate the length of the femoral tunnel. (**D**) The violet-marked line at the level of the femoral socket indicates that the femoral button has pulled out of the outer lateral femoral cortical by traction of both the white and blue sutures. (**E**) The femoral button is ready to be flipped by performing vigorous traction from the tibial end of the graft. (**F**) The graft is pulled into the femoral socket via alternate traction of the white sutures. LCF: lateral femoral condyle; PCL: posterior cruciate ligament; TP: tibial plateau.

The femoral shuttle suture is used through the AM portal to assist the passage of the femoral button of the adjustable-loop suspension device (UltraButton™, Smith&Nephew) through the femoral socket (Figure 9A,B).

A “flip-then-fill” technique is used [16] and the femoral button of the adjustable-loop suspension device is retrieved at the ACL femoral footprint level via traction of both the white and blue sutures of the femoral adjustable-loop suspension device. As soon as the previously violet-marked line on the femoral adjustable-loop suspension device (indicating the length of the femoral tunnel) becomes evident at the ACL femoral footprint level (Figure 9C,D), this will mean that the femoral button has pulled out of the outer lateral femoral cortical and is ready to be flipped by performing vigorous traction from the tibial end of the graft that is still outside the joint.

Once the femoral button has been flipped (Figure 9E), the graft is vigorously pulled into the femoral socket via traction of only the white sutures of the femoral adjustable-loop suspension device alternately (Figure 9F).

**Figure 10:**
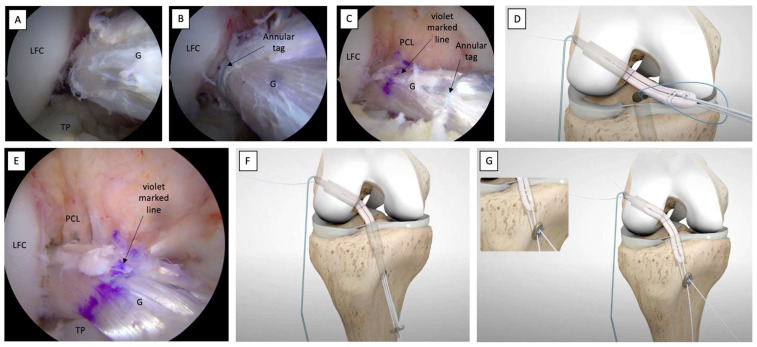
Complete graft passage into the femoral socket, with subsequent movement into the tibial socket and tensioning. (**A**,**B**) The graft progression inside the femoral socket is confirmed by the position of the annular tag located at the femoral graft edge. (**C**) The graft is ready to be carried into the tibial socket. The violet-marked line indicates the precise halfway point of the graft, while the annular tag in the tibial graft edge allows for better adjustment during the graft-pull maneuver under arthroscopic visualization. (**D**,**E**) The tibial button is pulled through the tibial socket by pulling the tibial shuttle sutures. (**F**) Traction of the white sutures alternately is begun. (**G**) The tibial button is secured in contact with the tibial cortical. LCF: lateral femoral condyle; G: graft; PCL: posterior cruciate ligament; TP: tibial plateau.

**Tips.**  *It may be helpful to partially position the femoral side of the graft at the level of the femoral socket and then pass the graft to the tibial socket so that the graft depth in the femoral socket may be better accommodated during tensioning. Usually, during tensioning of the femoral graft side, only 1 to 1.5 cm of the graft is pulled into the femoral socket. The graft progression inside the femoral socket is confirmed by the position of the annular tag at the femoral graft edge (Figure 10A,B).*

**Tips.**  *Proper flipping of the femoral button may be checked by direct arthroscopic viewing, using the AL portal as the entry point and positioning the scope at the lateral subcutaneous layer.*

### 2.10. Tibial Socket Graft Passage and Tensioning

The tibial button of the adjustable-loop suspension device (UltraButton™, Smith&Nephew) is passed through the tibial socket by pulling the tibial shuttle sutures (Figure 10C,D).

**Tips.**  *A multi-strand high-strength suture (FiberWire^®^ No. 0; Arthrex), placed 15–20 mm from the extremity of the graft and tagged in the middle of the graft with an annular mark, allows for better adjustment during the graft pull maneuver at both the femoral and tibial sockets under arthroscopic visualization.*

**Tips.**  *If an internal brace is used, consisting of a suture tape such as FiberTape^®^ (Arthrex), the free tails of the suture tape should be retrieved along with the sutures of the tibial adjustable-loop suspension device.*

After ensuring that the tibial graft side has been appropriately engaged in the tibial socket, the surgeon begins to pull the white sutures of the tibial adjustable-loop suspension device alternately, to bring the tibial button in contact with the tibial cortical (Figure 10E–G).

**Figure 11:**
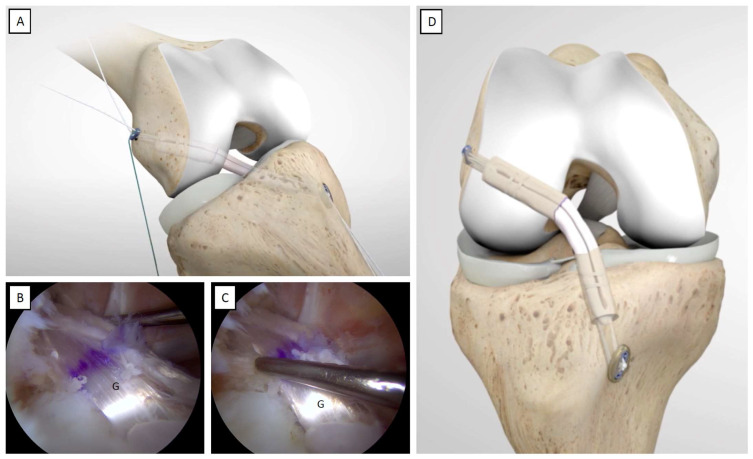
Cycling the knee, probing the graft, and completing of the all-inside ACLR. (**A**) Cycling of the knee is performed in complete flexion and extension movement. (**B**,**C**) The graft’s tension is tested using a probe. (**D**) The femoral and tibial buttons are properly fixed, the graft is in place, and the all-inside ACLR is completed. G: graft.

The tibial button should be fixed while keeping the knee in extension/hyper-extension and neutral rotation. The interposition of soft tissue between the tibial cortical and the tibial button during the tensioning phase should be avoided (Figure 11A).

Cycling of the knee in flexion–extension through its range of motion (ROM) is performed to help accommodate the graft. Further tension is applied at this point by manually pulling the white sutures of the femoral adjustable-loop suspension device. An arthroscopic examination of the successful tension and tightness of the graft positioned in its anatomical site is performed using a probe (Figure 11B,C).

After graft tensioning, the knee should be checked to ensure that full ROM is available. The white sutures of the tibial button are then tied at the top of the button and the remaining tails are cut off. At the femoral level, the white sutures of the femoral button may be secured using a knot-pusher under arthroscopic subcutaneous viewing, entering from the AL portal and placing the scope at the lateral subcutaneous layer, and, finally, the remaining tails are cut off (Figure 11D).

Finally, layer suturing is performed at the graft harvest site, using No. 0 Vicryl sutures, along with skin suturing of the arthroscopic portals and the graft harvest area with No. 2-0 Prolene sutures.

**Tips.**  *An excessively lengthy graft will dimple on the femoral or tibial socket floor and is, therefore, unacceptable since it fails to maintain proper graft tension.*

**Tips.**  *To promote greater tibial button–tibial bone contact and in cases where there is the suspicion of having maintained a short bone bridge at the level of the tibial socket, it is advisable to use an XTENDOBUTTON Fixation Device (Smith&Nephew), which has been designed to provide an extended tibial surface area of contact.*

**Tips.**  *If an internal brace (suture tape) is used, it must be held under tension during the tibial button tensioning maneuver and must then be tied and stabilized at the level of the tibial button by passing the extremities of the suture tape tails through the holes freed by the removal of the blue carrier sutures, or they could be anchored at the tibial level using fully threaded knotless SwiveLock (Arthrex) anchors, which have been designed for use with suture tape. During the internal brace fixation process, it becomes imperative to meticulously administer an equivalent or slightly reduced tension compared to the graft. This precautionary measure is crucial to prevent stress shielding.*

**Tips.**  *In case the graft appears loose after performing proper tensioning in the tibial site, it is recommended to perform the following procedures in this order:*

(1) *Check the amount of graft in the tibial socket according to the position of the annular tag at a predetermined distance from both ends of the graft and the annular tag in the middle of the graft.*
(2) *Tighten the femoral side further by approximately 5 mm.*
(3) *Perform additional cycling of the knee.*
(4) *Remove the graft from the tibial socket and repeat the retrograde reaming by increasing the drilling by 5 mm, put the shuttle suture back in place, retrieve it without soft tissue bridge interposition, and proceed with graft passage and tensioning.*

## 3. Discussion

This study’s main finding is that the all-inside ACLR technique represents a feasible procedure that may be performed in all patients with ACL injury, including adolescents with open physis, reporting clinical and functional outcomes that are in line with other ACLR techniques [4,18].

### 3.1. Patient-Reported Outcome Measures (PROMs)

Encouraging results were reported in the literature regarding the PROMs of patients undergoing ACLR using the all-inside technique; in general, similar or superior functional outcomes and a reduction in postoperative pain were described, compared with other ACLR techniques [8,18]. In their systematic review, Bhimani et al. [18] analyzed a cohort of 923 patients who underwent ACLR via the transportal (TP) technique and 1678 patients who underwent the all-inside ACLR technique. While an ST graft was predominantly employed in the all-inside ACLR technique group, a combined graft of ST and gracilis was the norm in the TP technique group. Postoperative enhancement of the International Knee Documentation Committee (IKDC) scores, Lysholm scores, KT-1000 measurements, and Short Form-12 (physical and mental) scores exhibited similar trends in both groups. Conversely, the visual analog scale (VAS) pain score was notably lower in the all-inside ACLR technique group than in the TP technique group. Furthermore, the rates of complications demonstrated parity between the two cohorts, and prospective comparative studies of the two techniques showcased fewer minor complications in the all-inside ACLR technique group than in the TP technique group. The rehabilitation protocol of the all-inside ACLR technique is based immediately after obtaining full knee extension; jogging is allowed 2 to 4 months after surgery, and a return to cutting and rotational sports is permitted 6 to 9 months after surgery. Bhimani et al. [18] showed in their systematic review that patients in the all-inside ACLR technique group demonstrated an average sport return time of 7 months (range: 4–12.5), contrasting with the sport return time of patients in the group treated with the TP technique, who averaged 8.3 months (range: 6–12).

Schurz et al. [10] reported a significantly higher IKDC score, Lysholm score, and Tegner activity scale postoperatively compared to preoperatively at 24 months of clinical follow-up in those patients who underwent the all-inside ACLR technique; additionally, the pain score, according to the VAS, was significantly lower at 3, 6, 12, and 24 months. Lubowitz et al. [11] prospectively compared the results of the all-inside ACLR technique with those of the “full tibial tunnel” technique, describing similar postoperative clinical, functional, and knee stability outcomes, except for the VAS pain score, which was lower for the all-inside ACLR technique at a mean follow-up of 2 years. In their study, Placella et al. [9], analyzed adolescent patients undergoing the all-inside ACLR technique with a minimum follow-up of 8 years, reporting an excellent return to sports rate, which was evaluated with the Tegner activity scale. Galan et al. [12] analyzed the clinical outcomes at a 5-year follow-up in young and high-demand sports patients who underwent the all-inside ACLR technique with the quadriceps tendon; the authors reported good to excellent functional results with low complication rates. Furthermore, in their study, Lowenstein et al. [19] exhibited good outcomes at the 1-year and 2-year follow-up stages following the procedure in patients who underwent a quadrupled ST graft with a minimum diameter of 9 mm. Lastly, Brzezinski et al. [20] recently documented the utilization of a 5-strand ST graft, showcasing biomechanical strength on a par with that of a quadrupled ST graft. Their findings suggest that this could be a viable alternative, should the final graft diameter fall short.

### 3.2. Advantages of the All-Inside ACLR Technique

The all-inside ACLR technique allows anatomical graft placement at the tibial and femoral sides, providing physiological benefits for graft revascularization and ligamentization [4]. This technique offers numerous advantages in the femoral half tunnel creation: it is performed independently from the tibial tunnel location, the outside-in aimer arm allows for femoral interosseous distance measurement before socket creation using specific sleeves [14], and, finally, this procedure is performed with the knee positioned at 90° of flexion, allowing optimal native ACL center visualization and easier outside-in aimer arm insertion through the AL portal. Compared with the trans-tibial approach, the all-inside ACLR technique allows anatomical femoral tunnel positioning because it is independent of the tibial one; moreover, relative to the transportal technique, the absence of hyperflexion position simplifies proper ACL native center identification [18,21]. The all-inside ACLR technique has benefited over the years from numerous hardware developments, such as retrograde drills and cortical suspension fixation device evolution. First-generation cortical fixation systems have rings of fixed length. In contrast, second-generation systems have adjustable-length rings so that, after flipping the button onto the outer cortex, the ring may be tightened by pulling the graft into the socket to fill the tunnel with the graft completely. Furthermore, first-generation cortical fixation buttons were designed for femoral fixation, while second-generation adjustable loop buttons are also effective for tibial fixation [7,14].

The all-inside ACLR technique allows for various graft choices, such as the autologous hamstring, quadriceps tendon, peroneus longus, and allografts. With double cortical suspension fixation, the necessary graft length is only about 6–7 cm; therefore, for example, the required length of 26–28 cm for graft preparation may be achieved with only ST [14]. Conte et al. [15], in their systematic review, reported that a tendon graft thickness of greater than 8 mm had lower failure rates in ACLR, and in the all-inside ACLR technique, this thickness could only be achieved using a quadrupled ST graft in most cases. However, many factors may influence graft thickness, such as age, sex, and height [15]. Therefore, it should be considered that not all patients in whom the ST is harvested could provide an ideal graft length and thickness. In these cases, the gracilis should be harvested secondarily to achieve an acceptable thickness and length [7,14,15]. Saving the gracilis may result in a smaller surgical incision and less donor-site morbidity [15]. Additionally, the hamstrings act as both knee flexors and tibial internal rotators; different studies demonstrated that gracilis preservation may lead to improved knee flexion strength, which could be helpful in activities or sports that require high hamstring strength [15,21,22,23,24].

Using half instead of complete tunnels, which preserves bone stock, is another advantage of the all-inside ACLR technique, especially in ACLR revision or multiple ligament reconstructions. Moreover, postoperative pain and swelling may be reduced since this technique does not violate the extra-articular cortex and periosteum. Several authors [14,18,25,26,27], using computed tomography (CT) studies, have emphasized the importance of bone stock preservation in orthopedic surgery [25], a condition that in ACLR is adequately achieved with the all-inside technique. Specifically, the authors [14,18,26,27] evaluated X-rays and CT scans of the socket with the all-inside ACLR technique, demonstrating a lesser socket widening and a better-preserved bone stock when compared to complete tunnels in other ACLR procedures. Bhimani et al. described a lower tibial plateau fracture incidence in the postoperative period due to cortical bone preservation and improved graft-to-bone integration since the “dead space” was minimal, with tibial socket creation instead of complete tunnels [18].

Lastly, in the all-inside ACLR technique, an independent femoral guide facilitates precise anatomical placement on the femoral ligament footprint, heightening the probability of reinstating the knee’s natural kinematics. Furthermore, it mitigates potential hazards, including forming an excessively short tunnel, posterior wall blowout, and encroachment upon the lateral wall of the intercondylar notch, which instead represents significant issues to be considered with other ACLR techniques [28].

### 3.3. Internal Brace Augmentation

Internal brace application in ACLR surgery is a relatively recent concept. Mackay et al. [29] were the first to describe employing a synthetic suture tape (FiberTape^®^, Arthrex) to reinforce ACLR. Subsequently, several authors [15,16,30] reported a suture reinforcement application to augment ACLR, specifically in the all-inside ACLR technique. The internal brace protects and prevents graft-thinning during the healing and remodeling stages. Additionally, it also acts as a secondary knee stabilizer, preventing graft ruptures or elongation over time, and gives thickness in a small graft diameter (<8 mm) [16]. During fixation, it is necessary to carefully apply an equal or slightly lower tension than that of the graft to avoid stress shielding [31]. In a recent systematic review, Zheng et al. [31] included 314 patients who underwent an all-inside ACLR technique with the internal brace. An internal brace-associated ACLR showed a better Tegner activity scale and a trend toward a significantly higher return to sport rates than isolated ACLR. Furthermore, in patients undergoing augmentation with an internal brace, postoperative improvements were observed, although they were not statistically significant, in the Lysholm score, IKDC, and KOOS scores, with good knee stability restoration at a mean follow-up of 16.7 months [31].

### 3.4. The All-Inside ACLR Technique’s Complications and Drawbacks

The all-inside ACLR technique is complex, and orthopedic surgeons must be aware of the associated pitfalls. This technique’s overall complication rate is 5.89% [18,21]; in detail, it includes graft failures (2.47%), knee extension loss from 5° to 10° and from 1° to 3° (0.76% and 0.38%, respectively), significant knee flexion loss (15°, 0.19%), cartilage or meniscal injuries (0.76%), knee anterior region hypoesthesia (0.38%), deep and superficial infections (0.38% and 0.19%, respectively), postoperative hematomas (0.19%), cyclops syndrome (0.19%), and retrograde drill bit breakage (0.19%) at a 2-year follow-up [16,31,32]. Finally, suspension fixation devices increase the risk of tunnel widening due to the “windshield wiper” effect [18,21].

The all-inside ACLR technique also has some drawbacks. The learning curve of some procedures, such as graft preparation, drilling, and socket creation, is more complex and challenging compared to other ACLR techniques [33]; for this reason, the operative times for the all-inside ACLR technique are usually longer than those of other ACLR procedures.

## 4. Conclusions

The all-inside ACLR technique was developed to improve ACLR surgical outcomes. This technique offers advantages such as less invasive surgery, reduced bleeding, faster recovery, and better graft tension control. Half, instead of complete, tunnel creation implemented quadrupled ST or quadriceps tendon as the grafts of choice. The all-inside ACLR technique applies to all patients with an ACL injury, including adolescents, and provides good clinical outcomes postoperatively, with medium- to long-term graft survival. Technique standardization may help avoid the potential complications associated with this procedure.

## Data Availability

The data presented in this study are available in the article.
